# Deep learning-assisted diagnosis of benign and malignant parotid tumors based on ultrasound: a retrospective study

**DOI:** 10.1186/s12885-024-12277-8

**Published:** 2024-04-23

**Authors:** Tian Jiang, Chen Chen, Yahan Zhou, Shenzhou Cai, Yuqi Yan, Lin Sui, Min Lai, Mei Song, Xi Zhu, Qianmeng Pan, Hui Wang, Xiayi Chen, Kai Wang, Jing Xiong, Liyu Chen, Dong Xu

**Affiliations:** 1 Department of Diagnostic Ultrasound Imaging & Interventional Therapy, Zhejiang Cancer Hospital, Hangzhou Institute of Medicine (HIM), Chinese Academy of Sciences, 310022 Hangzhou, Zhejiang China; 2 Postgraduate training base Alliance of Wenzhou Medical University (Zhejiang Cancer Hospital), 310022 Hangzhou, Zhejiang China; 3 Zhejiang Provincial Research Center for Cancer Intelligent Diagnosis and Molecular Technology, 310022 Hangzhou, Zhejiang China; 4 Wenling Big Data and Artificial Intelligence Institute in Medicine, 317502 TaiZhou, Zhejiang China; 5 Taizhou Key Laboratory of Minimally Invasive Interventional Therapy & Artificial Intelligence, Taizhou Campus of Zhejiang Cancer Hospital (Taizhou Cancer Hospital), 317502 Taizhou, Zhejiang China; 6 Second Clinical College, Zhejiang University of Traditional Chinese Medicine, 310022 Hangzhou, Zhejiang China; 7 Dongyang Hospital Affiliated to Wenzhou Medical University, 322100 Jinhua, Zhejiang China; 8 Shenzhen Institute of Advanced Technology, Chinese Academy of Sciences, 518000 Shenzhen, Guangdong China

**Keywords:** Deep learning, Parotid tumor, Ultrasound, Model-assisted

## Abstract

**Background:**

To develop a deep learning(DL) model utilizing ultrasound images, and evaluate its efficacy in distinguishing between benign and malignant parotid tumors (PTs), as well as its practicality in assisting clinicians with accurate diagnosis.

**Methods:**

A total of 2211 ultrasound images of 980 pathologically confirmed PTs (Training set: *n* = 721; Validation set: *n* = 82; Internal-test set: *n* = 89; External-test set: *n* = 88) from 907 patients were retrospectively included in this study. The optimal model was selected and the diagnostic performance evaluation is conducted by utilizing the area under curve (AUC) of the receiver-operating characteristic(ROC) based on five different DL networks constructed at varying depths. Furthermore, a comparison of different seniority radiologists was made in the presence of the optimal auxiliary diagnosis model. Additionally, the diagnostic confusion matrix of the optimal model was calculated, and an analysis and summary of misjudged cases’ characteristics were conducted.

**Results:**

The Resnet18 demonstrated superior diagnostic performance, with an AUC value of 0.947, accuracy of 88.5%, sensitivity of 78.2%, and specificity of 92.7% in internal-test set, and with an AUC value of 0.925, accuracy of 89.8%, sensitivity of 83.3%, and specificity of 90.6% in external-test set. The PTs were subjectively assessed twice by six radiologists, both with and without the assisted of the model. With the assisted of the model, both junior and senior radiologists demonstrated enhanced diagnostic performance. In the internal-test set, there was an increase in AUC values by 0.062 and 0.082 for junior radiologists respectively, while senior radiologists experienced an improvement of 0.066 and 0.106 in their respective AUC values.

**Conclusions:**

The DL model based on ultrasound images demonstrates exceptional capability in distinguishing between benign and malignant PTs, thereby assisting radiologists of varying expertise levels to achieve heightened diagnostic performance, and serve as a noninvasive imaging adjunct diagnostic method for clinical purposes.

**Supplementary Information:**

The online version contains supplementary material available at 10.1186/s12885-024-12277-8.

## Background

Parotid tumors (PTs) are the most prevalent neoplasms of the salivary glands, with a malignancy rate of 20% [1, 2]. Currently, surgical resection remains the primary treatment modality for PTs; However, different histological subtypes necessitate distinct surgical approaches and prognostic evaluations. Malignant parotid tumors (MPT) require more aggressive surgical techniques such as total parotidectomy [3, 4]. The fine needle aspiration cytology (FNAC) is the most commonly used qualitative method for preoperative diagnosis of PTs [5]. However, due to the extensive cellular heterogeneity and overlapping characteristics among various subgroups, it poses challenges in accurately diagnosing PTs [6]. Meanwhile, FNAC carries the risk of inducing inflammations and causing local tumor spread [7, 8]. Therefore, it is crucial to develop noninvasive and accurate methods for evaluating benign parotid tumors(BPT) and MPT prior to surgery in order to guide treatment decisions.

Ultrasound (US), computed tomography (CT), and magnetic resonance imaging (MRI) are commonly utilized for the assessment of parotid gland lesions, including positioning, diagnosis, and treatment evaluation. The clinical utility of MRI and CT in the assessment of patients is constrained by their high cost or potential for radiation exposure. In contrast, US has become the preferred imaging modality for parotid masses due to its simplicity, cost-effectiveness, and lack of radiation [9]. Nevertheless, the accuracy of these conventional imaging methods in the diagnosis of PTs is limited [10], and the actual prediction remains unsatisfactory. A meta-analysis of 38 studies involving 2753 patients with PTs demonstrated that the sensitivity of US, CT, and MRI in distinguishing between benign and malignant salivary gland tumors was found to be 66%, 70%, and 80% respectively [11]. Hence, there is a need to develop more effective imaging evaluation methods for histological classification of PTs.

The field of medical image analysis has witnessed a surge in attention towards deep learning(DL) in recent years. As a subset of machine learning, DL models employ multilayer neural networks for automatic feature extraction. By exploring high-dimensional data abstraction, these models effectively reduce the need for engineering-based characteristics [12–14]. DL-based models excel at extracting features from images that are imperceptible to the naked eye of radiologists, thereby greatly assisting in disease diagnosis. Convolutional neural networks (CNNs), as a prevalent DL method, show significant potential in the realm of medical images, especially based on US image [15–17]. At present, the DL model based on CT [18, 19] and MRI [20, 21] have been developed for the differential diagnosis of PTs. A recent study [22] utilized a 3D DenseNet-121 to construct a binary classifier capable of distinguishing PTs on arterial-phase enhanced CT images, however, the final model exhibited a specificity rate of only 66.7%. In another study [20], a DL model was constructed for distinguished MPT and BPT based on multi-parametric MRI images, however, the accuracy of the final model was low. To the best of our knowledge, the majority of previous studies have primarily relied on CT or MRI images for the identification of BPT and MPT. Nevertheless, due to inherent limitations associated with CT and MRI imaging modalities, the models derived from these investigations exhibited limited applicability. Simultaneously, only a few studies [23, 24] have explored DL techniques based on US images for distinguishing between BPT and MPT.

Therefore, the purpose of this study was to formulate a DL model based on US images, to verify its efficacy in discriminating BPT and MPT, and to compare the diagnostic performance of different radiologists with and without the assistance of the model. Additionally, an analysis of misclassified images by the DL model will be conducted to provide better guidance for clinical practice.

## Materials and methods

### Patients

The retrospective study was approved by the Ethics Committee of our Hospital and another Hospital, and informed consent was waived (IRB-2020-314). Retrospective collection of clinical and US imaging data was conducted on 1050 patients who underwent parotid gland surgery in two hospitals from February 2017 to May 2023.

**Fig. 1:**
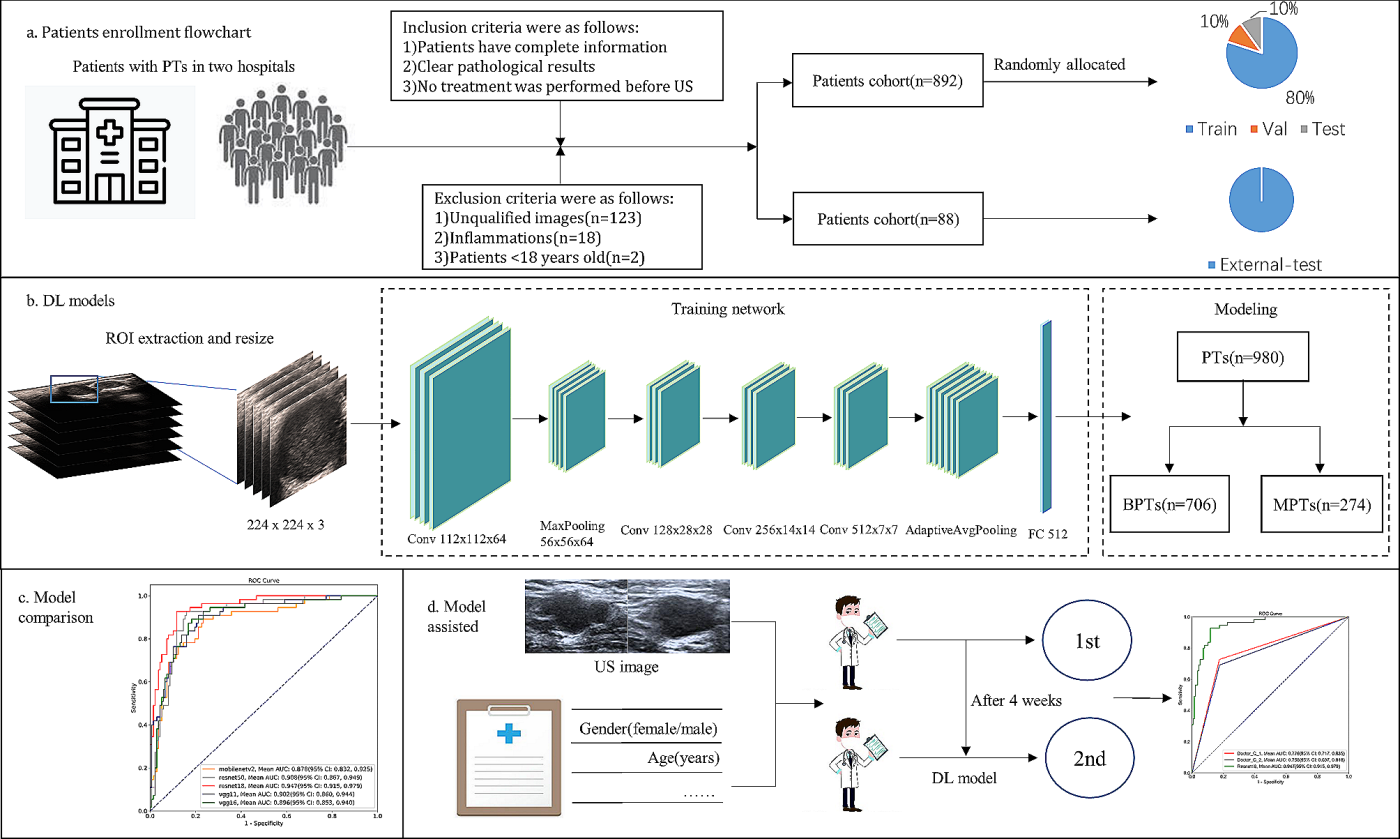
The overall pipeline of this study. (**a**) Flowchart of patient recruitment, the cohort of patients in our hospital was randomly divided into training set, validation set, and internal-test set, 88 patients from another hospital were assigned to a separate external-test set; (**b**) construction of five different DL models for identifying BPT and MPT based on US images; (**c**) comparison of diagnostic performance among different models using the AUC to select the best model in internal- and external- test set; (**d**) evaluation of whether radiologists with varying levels of experience show improved diagnostic performance with the assistance of the model

Inclusion criteria were as follows: (1) prior to the operation, all patients underwent US examination. (2) the histological type was confirmed through pathology, and complete clinical information was obtained. (3) no invasive procedures such as FNAC were performed before the US examination. Exclusion criteria were as follows: (1) poor image quality (motion artifacts or PTs not be fully visible due to attenuation/ mandible occlusion or PTs are much too large to be fully displayed); (2) inflammations lesions; (3) patients < 18 years old. Baseline clinical characteristics were extracted from the electronic health record, while histopathological data were retrieved from the Pathology Information Management System. A total of 980 PTs from 907 patients (Table S1 presents the distribution of histological diagnoses for all PTs) were included in the final cohort. Figure 1 illustrates the overall design flow diagram.

### US image acquisition

The patient was in the supine position, and the parotid mass underwent scanning using a conventional US scanner in both sagittal and transverse planes to obtain the complete image of lesions and their corresponding adjacent normal tissues. The Philips iU22 (ROYAL Philips; Amsterdam, the Netherlands), Esaote Mylab90 (Esaote S.P.A; Genoa, Provincia Di Genova, Italy), and Logic E9 (General Electric Company, Fairfield, Connecticut, USA) systems were utilized for ultrasonography assessment (Table S2 presents the distribution of different ultrasound devices in BPT and MPT). All scans were conducted with a linear array transducer operating at a broadband frequency range of 5–12 MHz. The entire set of images was considered, resulting in a final selection of 616 images and 260 patients for the MPT, as well as 1595 images and 647 patients for the BPT.

The following characteristics of the lesions were documented: Max-diameter, location (deep /superficial/both), Cystic areas (absent /present), composition (homogeneous /heterogeneous), margin (clear/unclear), shape (regular/irregular), posterior acoustic enhancement (absent /present), and calcification (absent /present). The US characteristics were qualitatively analyzed by two radiologists (radiologist A and B, with over 10 years of experience) who were blinded to the final histopathological findings. If there is a discrepancy, the US images will be reviewed by both radiologists until a consensus is reached. Interclass correlation coefficient (ICC) was used to assess inter-observer agreement in reading US features. ICC > 0.80 was considered excellent.

### Data pre-processing and segmentation

In this study, we utilized the OpenCV library in Python to convert the acquired US images from DICOM format to JPG format. and we manually removed any noise information present around the original image, such as patient’s name, the hospital name, the time of the examination, US equipment name, the body mark, equipment parameters, image numbers. Two radiologists (A and B) utilized Labelme software to manually delineate the tumor US images one by one and obtain rectangular regions of interest (ROI). To enable the model to capture more internal information and essential features within the images, we subsequently enlarged the delineated ROIs by 1.3 times before cropping the original images. The US images of PTs in our hospital were randomly divided into training, validation, and internal-test sets at an 8:1:1 ratio, and performed five-fold cross-validation on this dataset. Given the limited number of parotid datasets and the sparsity of features in medical data, the existing images underwent enhancements such as rotation (the maximum rotation angle is set to 15), flip (horizontal flip), scaling (maximum scale is set to 1), translation (maximum panning distance of -20 pixels to + 20 pixels), and mixed transformations to improve the generalization performance of the DL model. Additionally, to address variations in data resulting from different scanners, we applied histogram equalization to the existing images. The image length and width were adjusted from 1596 × 819 pixels to 224 × 224 pixels in accordance with the required input size of the model, followed by image normalization operation. We augment MPT image data and expand it until it matches BPT image data, which will be utilized for DL model training.

### Model establishment and validation

The study employed five distinct convolutional neural network models (Resnet18, Resnet50, Vgg11, Vgg16, Mobilenetv2) to extract features from BPT and MPT images and construct classification models. The model parameters were iteratively updated using the backpropagation method of the neural network to achieve the classification of BPT and MPT, and the best model was selected after comparing the AUC values. The final prediction for each nodule in the test cohort was calculated based on the aggregated results of all US images it contained. The soft voting method was employed to determine the average probability of malignancy for the nodule and generate the final prediction. Furthermore, we employed five-fold cross-validation to determine the final classification performance of the model by computing the average of the evaluation results from five runs. The diagnostic confusion matrix of the best model was obtained by comparing these predictions with histopathological results. Detailed training strategies can be found in the supplementary material.

### Subjective evaluation

We conducted two subjective evaluations to assess the auxiliary efficacy of the best DL model. Six radiologists, including two senior doctors (radiologists C and D with 22 and 18 years of experience respectively), two intermediate doctors (radiologists E and F with 11 and 10 years of experience respectively), as well as two junior doctors (radiologists G and H with 5 and 4 years of experience respectively), independently reviewed the internal-test set comprising US images, documenting their comprehensive interpretations of PTs (benign or malignant). While reviewing the US images, each radiologist was blinded to the final histopathological findings. Following a four-week buffer period, a different random order was adopted for DL readout of the model results (including classification outcomes and malignant probabilities) and reevaluation of the US images by radiologists. The diagnostic results of the radiologists were re-recorded to assess whether the diagnostic performance of the radiologists was enhanced when utilizing the DL model (Fig. 1.d).

### Statistical analysis

The baseline data of patients were subjected to statistical analysis using SPSS software (version 25.0, IBM). Python (version 3.8.15) was employed for model development and calculation of indicators in this study. Statistical significance was considered when *P* < 0.05. Further details regarding the statistical analysis can be found in the Supplementary Material.

## Results

### Baseline characteristics

Included in this study, 907 patients (male 542, female 365) of 980 cases of PTs, of which 260 patients were diagnosed with MPT, 647 patients were diagnosed with BPT, training cohort includes 1638 images from 721 PTs (MPT and BPT were 215, 506, respectively). The validation cohort included 194 images from 82 PTs (MPT, BPT were 25, 57, respectively), and the internal-test cohort included 192 images from 89 PTs (MPT, BPT were 25, 64, respectively), the external-test cohort included 187 images from 88 PTs (MPT, BPT were 9, 79, respectively). Mucoepidermoid carcinoma was the most prevalent pathological type in MPT (34.2%) and pleomorphic adenomas (PAs) in BPT (30.9%), followed by Warthin tumors (WTs) (26.5%). A detailed summary of radiographic characteristics among PTs groups is presented in Table 1. In the training cohort, significant differences were observed between BPT and MPT regarding age, shape, margin, posterior echogenicity, and calcification (*P* < 0.05). Maximum tumor diameter, composition, cystic areas did not show statistical significance (*P* > 0.05). Multivariate regression analysis revealed that irregular shape, unclear margins, and lack of posterior acoustic enhancement were associated with MPT (Supplementary Table 3). The Cohen Kappa test values for both radiologists A and B in the acquisition of US features were greater than 0.800(*P* < 0.001) (Supplementary Table 4).

**Table 1:** Baseline clinical-radiological features of the datasets

Variable		Training cohort(*n* = 721, image = 1638)			Validation cohort (*n* = 82, image = 194)			Internal-test cohort (*n* = 89, image = 192)			External-test cohort(*n* = 88, image = 187)		
		Benign (*n* = 506, Image = 1155)	Malignant (*n* = 215, Image = 483)	*p*-value	Benign (*n* = 57, Image = 136)	Malignant (*n* = 25, Image = 58)	*p*-value	Benign (*n* = 64, Image = 139)	Malignant (*n* = 25, Image = 53)	*p*-value	Benign (*n* = 79, Image = 165)	Malignant (*n* = 9, Image = 22)	*p*-value
Age (years)		56.00(44.00,64.00)	59.00(49.00,69.00)	0.001	57.00(41.00,69.00)	52.00(37.00,65.50)	0.372	56.50(43.75,63.00)	59.00(52.00,71.50)	0.203	65.00(54.00,75.00)	72.00(59.00,77.50)	0.380
Max-diameter (mm)		23.00(17.00,32.00)	24.00(18.00,32.00)	0.401	24.00(16.00,34.00)	22.50(15.00,27.20)	0.126	22.95(16.75,28.25)	26.00(17.00,38.70)	0.172	24.00(18.00,31.00)	24.00(16.00,76.00)	0.634
Sex	Female	186 (40.5%)	78 (38.6%)	0.644	24 (43.6%)	9 (36.0%)	0.520	20 (37.0%)	5 (20.8%)	0.157	41 (51.9%)	4 (44.4%)	0.736
	Male	273 (59.5%)	124 (61.4%)		31 (56.4%)	16 (64.0%)		34 (63.0%)	19 (79.2%)		38 (48.1%)	5 (55.6%)	
Location	Superficial	289 (63.0%)	113 (55.9%)	0.183	30 (54.5%)	17 (68.0%)	0.257	36 (66.7%)	16 (66.7%)	1.000	67 (84.8%)	8 (88.9%)	1.000
	Deep	166 (36.2%)	88 (43.6%)		25 (45.5%)	8 (32.0%)		18 (33.3%)	8 (33.3%)		12 (15.2%)	1 (11.1%)	
	Both	4 (0.9%)	1 (0.5%)		0 (0%)	0 (0%)		0 (0.0%)	0 (0.0%)		0 (0.0%)	0 (0.0%)	
Shape	Regular	365 (72.1%)	87 (40.5%)	<0.001	41 (71.9%)	12 (48.0%)	0.037	48 (75.0%)	8 (32.0%)	0.004	77 (97.5%)	2 (33.3%)	<0.001
	Irregular	141 (27.9%)	128 (59.5%)		16 (28.1%)	13 (52.0%)		16 (25.0%)	17 (68.0%)		2 (2.5%)	6 (66.7%)	
Margin	Clear	491 (97.0%)	102 (47.4%)	<0.001	53 (93.0%)	9 (36.0%)	<0.001	64 (100.0%)	4 (16.0%)	<0.001	79 (100.0%)	6 (66.7%)	0.001
	Unclear	15 (3.0%)	113 (52.6%)		4 (7.0%)	16 (64.0%)		0 (0.0%)	21 (84.0%)		0 (0.0%)	3 (33.3%)	
Echo	Homogeneous	103 (20.4%)	42 (19.5%)	0.801	11 (19.3%)	5 (20.0%)	1.000	10 (15.6%)	1 (4.0%)	0.171	38 (48.1%)	2 (22.2%)	0.174
	Heterogeneous	403 (79.6%)	173 (80.5%)		46 (80.7%)	20 (80.0%)		54 (84.4%)	24 (96.0%)		41 (51.9%)	7 (77.8%)	
Cystic areas	Absent	424 (83.8%)	177 (82.3%)	0.628	51 (89.5%)	20 (80.0%)	0.297	61 (95.3%)	23 (92.0%)	0.617	60 (79.5%)	5 (55.6%)	0.058
	Present	82 (16.2%)	38 (17.7%)		6 (10.5%)	5 (20.0%)		3 (4.7%)	2 (8.0%)		19 (24.1%)	4 (44.4%)	
Posterior acoustic enhancement	Absent	55 (10.9%)	114 (53.0%)	<0.001	10 (17.5%)	11 (44.0%)	0.012	6 (9.4%)	20 (80.0%)	<0.001	3 (3.8%)	5 (55.6%)	<0.001
	Present	451 (89.1%)	101 (47.0%)		47 (82.5%)	14 (56.0%)		58 (90.6%)	5 (20.0%)		76 (96.2%)	4 (44.4%)	
Calcification	Absent	486 (96.0%)	189 (87.9%)	<0.001	54 (94.7%)	22 (88.0%)	0.363	61 (95.3%)	19 (76.0%)	0.013	77 (97.5%)	5 (55.6%)	0.001
	Present	20 (4.0%)	26 (12.1%)		3 (5.3%)	3 (12.0%)		3 (4.7%)	6 (24.0%)		2 (2.5%)	4 (44.4%)	

### Performance of DL models

**Fig. 2:**
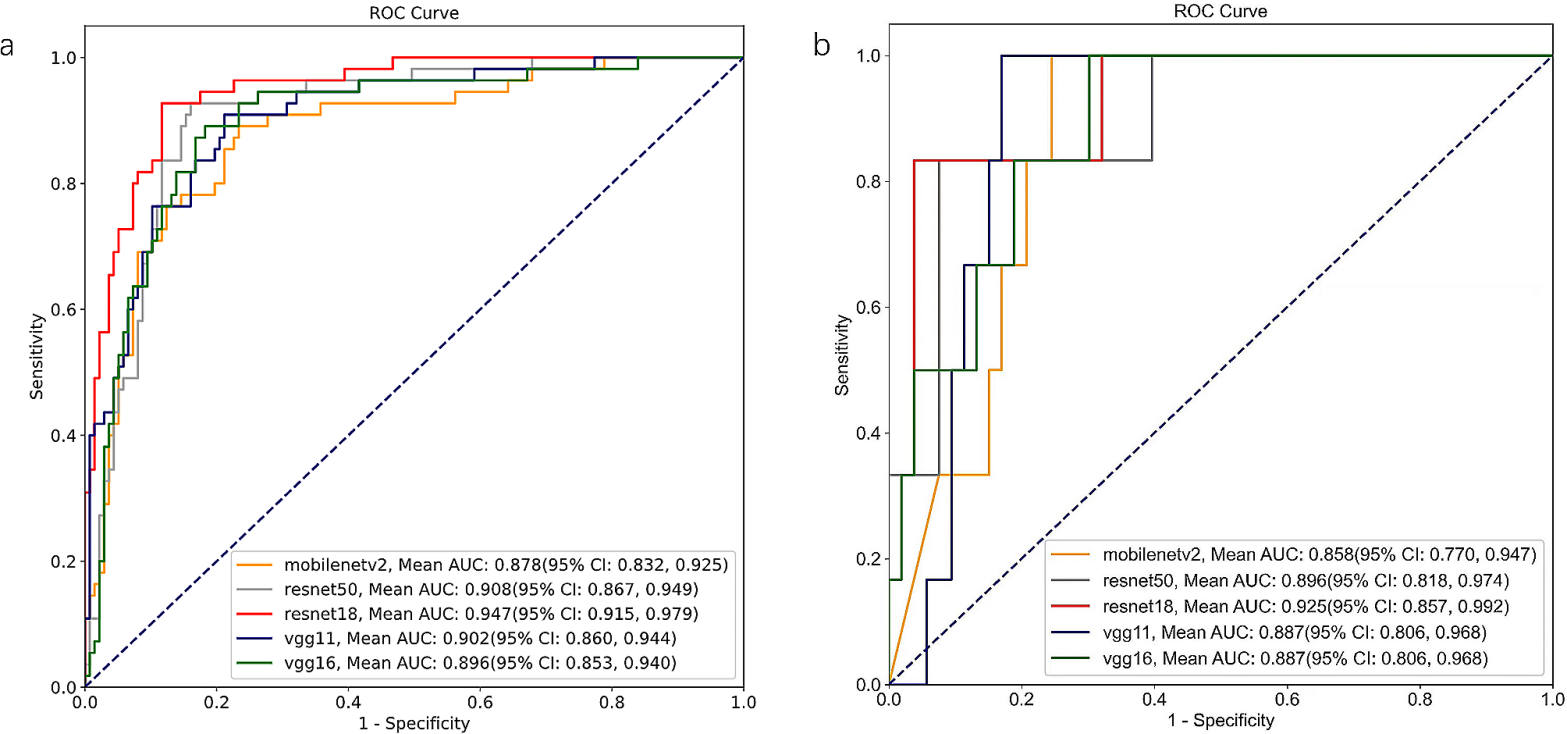
The receiver operating curves of various DL models in the internal- and external-test set. (**a**) internal-test set. (**b**) external-test set

The results presented in Fig. 2 demonstrate the excellent performance of the DL model on the internal-test and external- set, as evidenced by the five types of DL ROC and their corresponding AUC values (Supplementary Fig. 1 show the loss versus epoch during CNN model training and validation). Specifically, Resnet18, Resnet50, Vgg11, Vgg16, and Mobilenetv2 achieved AUC values of 0.947[95% CI: 0.915,0.979], 0.908[95% CI: 0.867,0.979], 0.902[95% CI: 0.860,0.944], 0.896[95% CI: 0.866,0.948], 0.878[95% CI: 0.832,0.925] in the internal-test set, and 0.925[95% CI: 0.857,0.992], 0.896[95% CI: 0.818,0.974], 0.887[95% CI: 0.806,0.968], 0.887[95% CI: 0.806,0.968], 0.858[95% CI: 0.770,0.947] in the external-test set. Resnet18 demonstrated the highest diagnostic performance, achieving an accuracy of 88.5%, a sensitivity of 78.2%, and a specificity of 92.7% in the internal-test set. The model’s performance evaluation index in internal- and external-test set is presented in Table 2, with Delong analysis revealing statistically significant differences between Resnet18’s AUC value and those of other models (Supplementary Table 5).

**Table 2:** The performance comparison of different models in internal- and external- test set

Model		AUC (95% CI)	Accuracy	Sensitivity	Specificity	PPV	NPV	F1	Kappa
Resnet 18	Internal-test	0.947(0.915,0.979)	0.885(0.840, 0.930)	0.782(0.673, 0.891)	0.927(0.883, 0.971)	0.811(0.706, 0.917)	0.914(0.867, 0.960)	0.796	0.717
	External-test	0.925(0.857, 0.992)	0.898(0.821, 0.975)	0.833(0.535, 1.132)	0.906(0.827, 0.984)	0.500(0.190, 0.810)	0.980(0.940, 1.019)	0.625	0.570
Resnet 50	Internal-test	0.908(0.867,0.979)	0.797(0.740, 0.854)	0.491(0.359, 0.623)	0.920(0.874, 0.965)	0.711(0.566, 0.855)	0.818(0.757, 0.879)	0.581	0.452
	External-test	0.896(0.818, 0.974)	0.864(0.777, 0.952)	0.833(0.535, 1.132)	0.868(0.777, 0.959)	0.417(0.138, 0.696)	0.979(0.937, 1.020)	0.556	0.486
Vgg 11	Internal-test	0.902(0.860,0.944)	0.839(0.786, 0.891)	0.691(0.569, 0.813)	0.898(0.847, 0.949)	0.731(0.610, 0.851)	0.879(0.824, 0.933)	0.710	0.598
	External-test	0.887(0.806, 0.968)	0.847(0.756, 0.939)	0.833(0.535, 1.132)	0.849(0.753, 0.945)	0.385(0.120, 0.649)	0.978(0.936, 1.020)	0.526	0.450
Vgg 16	Internal-test	0.907(0.866,0.948)	0.839(0.786, 0.891)	0.764(0.651, 0.876)	0.869(0.812, 0.925)	0.700(0.584, 0.816)	0.902(0.851, 0.952)	0.730	0.616
	External-test	0.887(0.806, 0.968)	0.814(0.714, 0.913)	0.833(0.535, 1.132)	0.811(0.706, 0.917)	0.333(0.095, 0.572)	0.977(0.933, 1.021)	0.476	0.387
Mobilenetv2	Internal-test	0.878(0.832,0.925)	0.839(0.786, 0.891)	0.709(0.589, 0.829)	0.891(0.838, 0.943)	0.722(0.603, 0.842)	0.884(0.831, 0.937)	0.716	0.603
	External-test	0.858(0.770, 0.947)	0.797(0.694, 0.899)	0.667(0.289, 1.044)	0.811(0.706, 0.917)	0.286(0.049, 0.522)	0.956(0.895, 1.016)	0.400	0.300

AUC area under the curve, PPV positive prediction value, NPV negative prediction value

### Diagnostic performance of the Radiologist and deep learning model-assisted diagnosis

We analyzed radiologists’ composite interpretations of PTs in the first round (Table 3) in the internal-test set and compared them with the metrics of the DL model. The results demonstrate that the DL model diagnosis efficiency surpassed that of six radiologists, with a Resnet18 AUC of 0.947 (95% CI = 0.915–0.979). The AUC for senior doctors was 0.776 and 0.772, while it was 0.734 and 0.745 for intermediate doctors, and finally, it was found to be 0.591 and 0.616 for junior doctors.

**Fig. 3:**
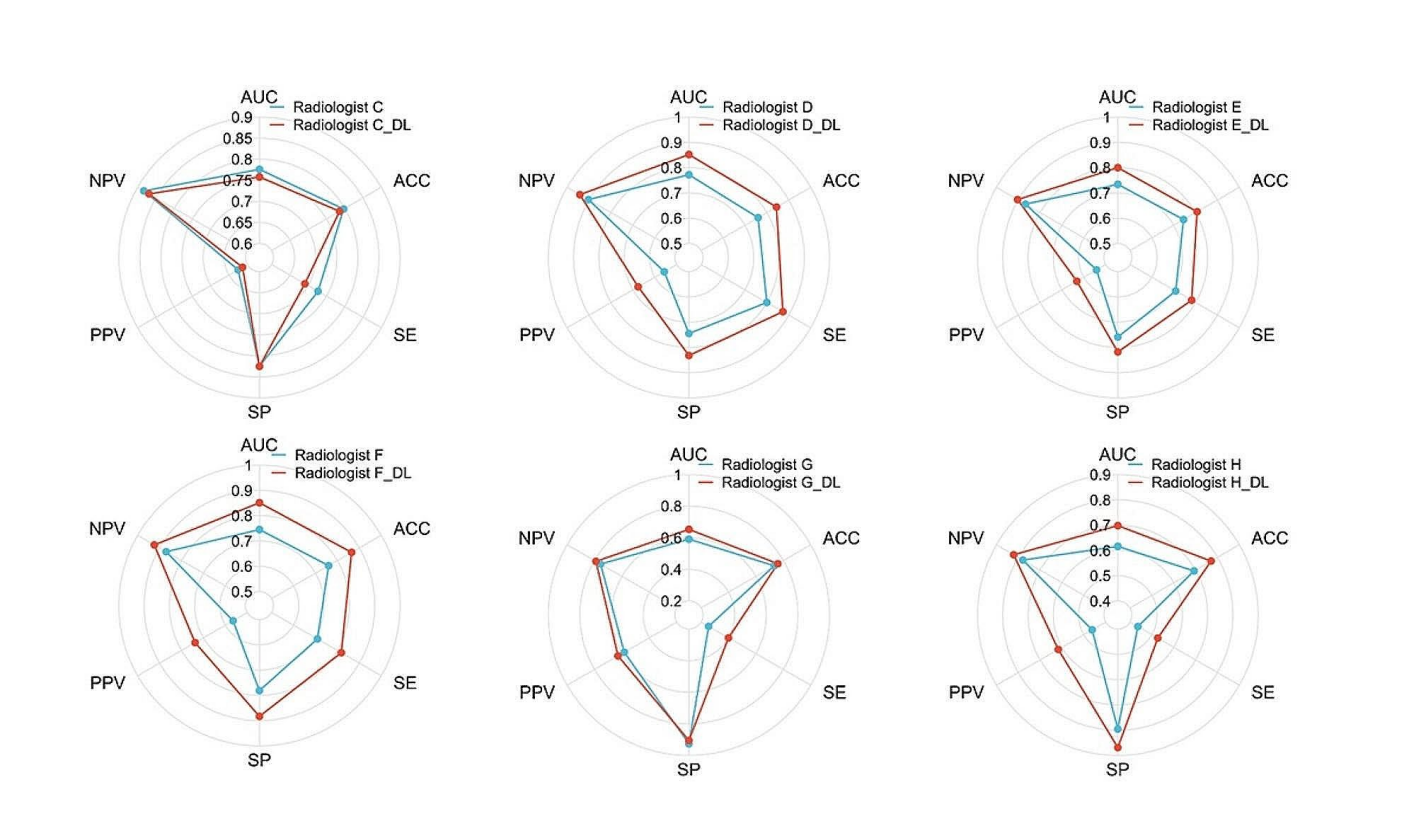
The radar chart illustrates the variations in each evaluation index during model-assisted diagnosis conducted by individual radiologists in the internal-test. AUC, area under the receiver operating characteristic curve, ACC, Accuracy. SE, Sensitivity. SP, Specificity. PPV, positive predictive value. NPV, negative predictive value

The subjective evaluation results of each radiologist in the second round were compared with those of the first round simultaneously. With the assistance of the model, most radiologists demonstrated improved diagnostic efficacy, resulting in an increased AUC value for radiologist D to 0.852. The AUC values for radiologist E and F also increased to 0.800 and 0.851 respectively, while radiologist G and H achieved increases to 0.653 and 0.698 respectively; however, there was a decrease in the AUC value for radiologist C to 0.758. Figure 3 illustrates the changes observed in each index evaluated subjectively by every radiologist during both rounds.

**Table 3:** Performance comparison between Resnet18 and radiologists without model assistance in the internal-test

	AUC (95% CI)	Accuracy	Sensitivity	Specificity	PPV	NPV	F1	Kappa
Senior								
Radiologist C	0.776(0.717,0.835)	0.797	0.727	0.825	0.625	0.883	0.672	0.526
Radiologist D	0.772(0.713,0.832)	0.760	0.800	0.745	0.557	0.903	0.657	0.482
Intermediate								
Radiologist E	0.734(0.672,0.797)	0.745	0.709	0.759	0.542	0.867	0.614	0.429
Radiologist F	0.745(0.683,0.807)	0.760	0.709	0.781	0.565	0.870	0.629	0.455
Junior								
Radiologist G	0.591(0.521,0.660)	0.734	0.255	0.927	0.583	0.756	0.354	0.218
Radiologist H	0.616(0.547,0.685)	0.693	0.436	0.796	0.462	0.779	0.449	0.236
Resnet 18	0.947(0.915,0.979)	0.885	0.782	0.927	0.811	0.914	0.796	0.717

AUC area under the curve, PPV positive prediction value, NPV negative prediction value

### Visual interpretation of the DL model

**Fig. 4:**
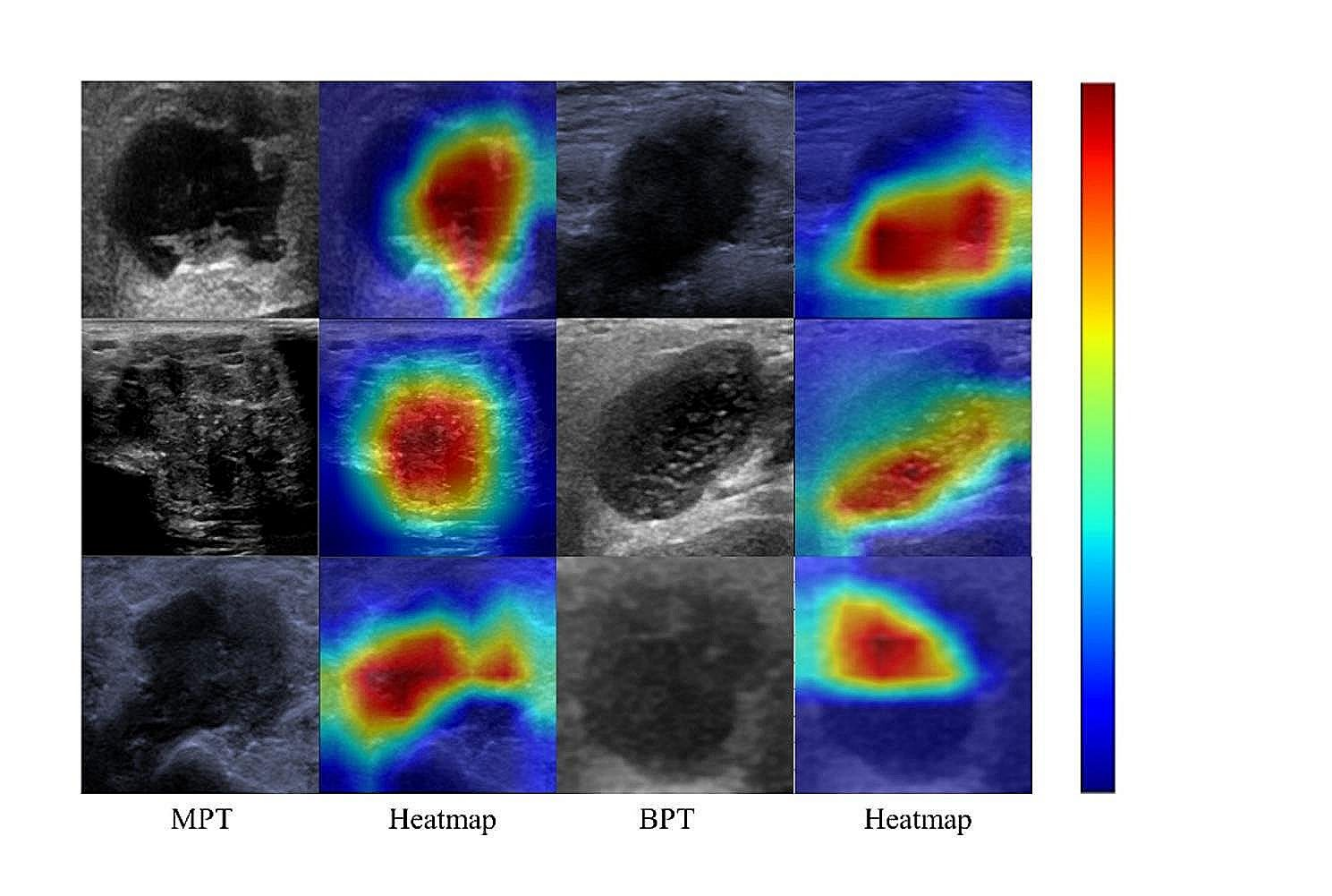
Visualization and interpretation of DL models in internal-test set. US images corresponding to BPT and MPT and their heat maps

The heat maps corresponding to the US images of BPT and MPT are given in Fig. 4. The different color distributions reflect the model’s focus on the most predictive regions of the US images. The red portion of the image provides crucial information for accurately determining the highlighted areas within the image model, thus aiding in prediction processes. The findings indicate that for accurately predicted parotid nodules, the red region depicted in the heat map is predominantly localized within the nodule itself; thus, enhancing the interpretability of the model through utilization of the heat maps.

### Analysis of misjudged pictures

**Fig. 5:**
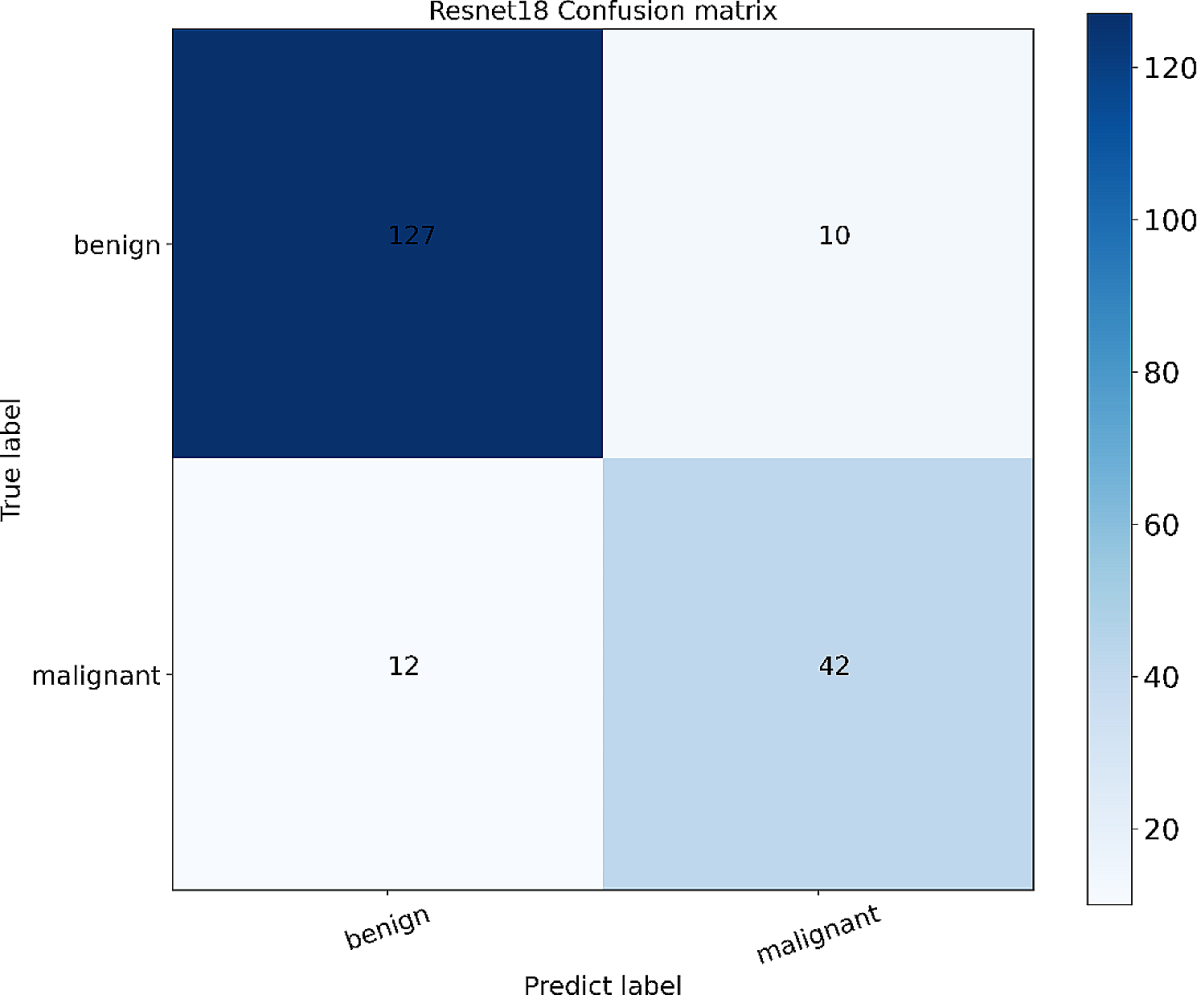
Diagnostic confusion matrix analysis was performed on the DL model; rows represent true labels and columns represent predicted labels. 10 of the BPT images were determined to have a malignant probability exceeding 0.5, while 12 of the MPT images had a malignant probability below or equal to 0.5

For each image in the internal-test set, the Resnet18 will integrate all the information in the ROI and finally obtain a probability, which is the probability that the nodule is considered as an MPT by the model. For multiple US images of the same nodule, we used a soft voting method to obtain the final prediction result for multiple US images of the same nodule. The threshold was set at 0.5, and the model classified the output as malignant when the probability exceeded 0.5, and as benign when the probability was less than or equal to 0.5. The final histopathology was compared with the model output, resulting in the selection of a total of 22 images. (Fig. 5 illustrates the diagnostic confusion matrix generated by the DL model). Table 4 displays the ultrasonographic characteristics of the nodules depicted in all 22 images.

**Table 4:** US characterization of PTs with model misjudgment

Variable		BPT(*n* = 10)	MPT(*n* = 12)
Location	Superficial	9 (90.0%)	8 (66.7%)
	Deep	1 (10.0%)	4 (33.3%)
	Both	0 (0.0%)	0 (0.0%)
Shape	Regular	2 (20.0%)	7 (58.33%)
	Irregular	8 (80.0%)	5 (41.67%)
Margin	Clear	10 (100.0%)	5 (41.67%)
	Unclear	0 (0.0%)	7 (58.33%)
Composition	Homogeneous	2 (20.0%)	2 (16.7%)
	Heterogeneous	8 (80.0%)	10 (83.3%)
Cystic areas	Absent	0 (0.0%)	4 (33.3%)
	Present	10 (100.0%)	8 (66.7%)
Posterior acoustic enhancement	Absent	7 (70.0%)	4 (33.3%)
	Present	3 (30.0%)	8 (66.7%)
Calcification	Absent	10 (100.0%)	10 (83.3%)
	Present	0 (0.0%)	2 (16.7%)

## Discussion

The present study involved the development and evaluation of five DL models for the noninvasive discrimination between MPT and BPT. The proposed DL model exhibited excellent diagnostic performance in distinguishing BPT from MPT, with the resnet18 achieving an impressive AUC of 0.947 in the internal-test set and 0.925 in the external-test set. The resnet18 has achieved a high AUC in assisting both senior and junior doctors, indicating its potential to enhance diagnostic performance for radiologists. Importantly, this study represents the first attempt at utilizing DL models for image analysis misjudgment.

In this study, we conducted a re-analysis of the model misjudgments in order to enhance their professional interpretation. Among the tumors that were incorrectly classified as MPT, it was observed that 80% were identified as PA (8/10), all exhibiting imaging characteristics consistent with malignant tumors such as heterogeneous composition and irregular lobulation. Conversely, tumors misclassified as BPT predominantly displayed regular shape without any cystic area or posterior acoustic enhancement. Consequently, it is imperative to exercise greater caution when interpreting discrimination results provided by the model in cases where similar US features are present in PTs.

The clinical information and US images in patients with differential diagnosis value remain a subject of controversy. In the training set, the multivariate logistic regression analysis revealed that age is not an independent predictor for distinguishing between BPT and MPT, which contradicts previous studies [24, 25]. At the same time, there was no significant difference in MPT and BPT incidence between men and women, indicating that gender cannot be used to assess the risk parameters of MPT. This conclusion aligns with the findings of Comoglu et al [26]. Our study also suggests that BPT typically exhibits a regular shape, well-defined edges, and enhanced posterior echo, which aligns with the findings of certain previous studies [10, 27–29]. However, owing to tissue heterogeneity, low-grade malignant tumors may also manifest benign tumor characteristics such as distinct boundaries [30], resulting in significant overlap in ultrasound features between BPT and MPT [31]. The use of other US techniques, such as acoustic elasticity imaging, has been reported for the differentiation of parotid benign and malignant diseases [32]. However, the utility of elasticity imaging in identifying MPT and BPT is limited. Currently, there is no consensus on PT imaging characteristics, thus necessitating the development of a more effective approach to assist in the identification of BPT and MPT.

The distinction between BPT and MPT has been previously established through the utilization of advanced CT, MRI-based radiomics, or DL methodologies [19, 20, 33–35]. Zheng et al. [18] extracted radiomics features from plain scan, arterial phase, and venous phase CT images of 388 patients. These features were combined with clinical characteristics to construct a joint model that achieved an AUC of 0.904 in the training set and 0.854 in the test set. The radiomics model developed by He et al [33] was based on morphological MRI images of 298 patients and aimed to differentiate MPT, PAs, WTs, and other benign tumors. However, its performance still surpasses that of radiologists (0.708 vs. 0.492). The Inception ResNetV2 model was established by Gunduz et al [20] in their study, utilizing multi-parametric MRI images, and the PTs were classified using the majority voting method, resulting in a final accuracy of 0.921. However, there is a limited adoption of DL models based on US images for distinguishing between these two tumors among scholars. Wang et al. [36] developed the EfficientNetB3 model using 251 PTs’ US images to preoperatively identify benign and malignant parotid gland lesions; however, the resulting AUC value was only 0.82, possibly due to the small sample size, indicating suboptimal performance of the trained model. The DL model was trained by Tu [24] using 638 US images, achieving a test set sensitivity of 100%. However, in this study, the training set for BPT and MPT images was manually selected to achieve a balanced ratio of 1:1, indicating evident selection bias (Supplementary Table 6). Our study included the largest sample size to date and employed five transfer learning models to accurately differentiate between BPT and MPT. The top-performing model achieved an AUC value of 0.947 in internal-test set and 0.925 in external-test set, indicating its potential as a clinically reliable imaging diagnostic tool.

In addition, the model’s classification results and malignant probability were presented to radiologists for diagnostic assistance. We conducted an analysis of radiologists’ reading results for the first time and discovered that the performance of radiologists with varying levels of experience was unsatisfactory. The mean AUC for senior, intermediate, and junior radiologists were only 0.774, 0.740, and 0.604 respectively, which may be attributed to the overlapping imaging features of PTs that cause confusion during visual assessment by radiologists and also due to the fact that we provided only static US images during evaluation. However, it is crucial to acknowledge that actual US examinations are dynamic processes and limited sections can lead radiologists to erroneous judgments. After the implementation of the diagnostic model, radiologists with varying levels of experience showed different degrees of improvement in their AUC. This demonstrates the extent to which the model we developed can assist radiologists of varying experience in identifying MPTs and BPTs. However, it is worth noting that one senior radiologist (radiologist C) did not observe improvements across all evaluation indices after utilizing the auxiliary diagnostic model. It is worth noting that despite Resnet18 achieving an AUC value of 0.947, no radiologist in the model has surpassed its performance by attaining higher AUC. May be due to excessive physician subjectivity or algorithmic aversion [37]. Previous studies [38] have compared the performance of multiple human experts assisted by artificial intelligence and concluded that highly skilled human experts are more prone to algorithm aversion, meaning they are less likely to accept suggestions from artificial intelligence.

The present study has several limitations: Firstly, it is a retrospective study conducted at two centers, which may introduce potential selection bias. Secondly, the number of misjudgment cases included in this study was limited, and therefore the results obtained from the analysis may not be entirely conclusive. Lastly, given its retrospective nature, further prospective studies are required to validate this system before its implementation in actual clinical practice. Addressing this issue will be a crucial focus for our future research.

## Conclusion

In conclusion, the research and development involved testing a DL auxiliary diagnostic model based on US images for the identification of BPT and MPT. The model exhibited excellent diagnostic performance, thereby enhancing the radiologist’s ability to provide accurate diagnoses. Additionally, we conducted an analysis of misclassification cases in DL models and summarize the distinguishing features of misclassified images, aiming to enhance clinical guidance and offer a potential approach for optimizing clinical treatment strategies.

### Electronic supplementary material

Below is the link to the electronic supplementary material.

Supplementary Material 1

## Data Availability

The datasets generated or analyzed during the study are not publicly available due to protect the privacy of patients but are available from the corresponding author on reasonable request.

## Abbreviations

DL: Deep learning
PTs: Parotid tumors
WTs: Warthin tumors
PAs: Pleomorphic adenomas
AUC: Area under curve
ROC: Receiver-operating characteristic
MPT: Malignant parotid tumors
FNAC: Fine needle aspiration cytology
BPT: Benign parotid tumors
US: Ultrasound
CT: Computed tomography
MRI: Magnetic resonance imaging
ROI: Regions of interest
DICOM: Digital Imaging and Communications in Medicine
NPV: Negative predictive value
PPV: Positive predictive value
ACC: Accuracy
SE: Sensitivity
SP: Specificity
