# Supplementary material for: Deep learning-assisted diagnosis of benign and malignant parotid tumors based on ultrasound: a retrospective study
Source: BMC Cancer. 2024 Apr 23;24:510. doi: 10.1186/s12885-024-12277-8 (PMC11036551; doi:10.1186/s12885-024-12277-8)
Supplement: Supplementary file 1 — Supplementary Material 1 [file 12885_2024_12277_MOESM1_ESM.docx]

**Supplementary Material**

**1.** **Details of DL model training strategy**

The transfer learning process involved utilizing the pre-trained weight parameters, where the fully connected layer from the original model was excluded and replaced with four additional fully connected layers. These added layers consisted of 1024, 512, 256, and 2 neurons respectively. The model underwent a total of 200 training epochs, with the first 100 epochs dedicated to freezing the model and the subsequent 100 cycles focused on unfreezing it for fine-tuning, aiming to expedite the discovery of the global optimal solution. The model was equipped with dropout regularization between the fully connected layers of the four-layer architecture to mitigate overfitting. During the training process, a random deactivation of 50% of neurons occurred. The model weight parameters were iteratively updated using binary cross entropy and the Adam optimizer. A dynamic adjustment strategy was incorporated, with an initial learning rate of 0.001 for the optimizer and the training process utilized a batch size of 8.

The SoftMax activation function was employed to generate the probability of benign and malignant parotid gland tumors, enabling the deep learning model to discern between benign and malignant parotid tumors. These five models extracted parotid image features using convolutional neural networks with distinct structures and depths, facilitating the classification of benign and malignant parotid glands through backpropagation and optimization algorithms.

**2. statistical analysis**

The continuous variables were reported as mean ± standard deviation or median (interquartile range [IQR]) after assessing normality using the Shapiro-Wilk test, and categorical variables are expressed as percentage. Student’s t-test or the Mann-Whitney U test was applied for differences analyzed of continuous variables, and the chi-square test or Fisher’s exact test was used for categorical variables. Variables with a p-value less than 0.20 in the univariate analysis were included in the multivariate logistic regression analysis with positive selection.

The model in this study is established and the calculation of each index is performed using Python (version 3.8.15). The model is built on pytorch (version 1.9.0), and scikit-learn (version 1.2.2) is utilized for dataset partitioning. Numpy (version 1.21.5) is employed for ROC analysis to calculate the AUC value of each model, as well as ACC, SE, SP, NPV, PPV, F1-score for evaluating the models' performance. Additionally, the DeLong test is applied to assess differences between AUCs when comparing different seniority radiologists' diagnostic efficiency with the assistance of the model; a P-value less than 0.05 indicates statistical significance. Cohen Kappa test for evaluating inter-observer agreement. ICC >0.80 was considered excellent.

Table S1. Histologic analysis of parotid tumors

| Histologic type |  | Patients (n) | % |
| --- | --- | --- | --- |
| Benign tumors |  | 647 | 71.3 |
| Pleomorphic adenoma |  | 280 | 30.9 |
| Warthin tumor |  | 240 | 26.5 |
| Basal cell adenoma |  | 110 | 12.1 |
| Other tumors |  | 17 | 1.8 |
| Malignant tumors |  | 260 | 28.7 |
| Mucoepidermoid carcinoma |  | 89 | 9.8 |
| Acinic cell carcinoma |  | 45 | 5.0 |
| Lymphoma/lymphoepithelial Carcinoma |  | 36 | 4.0 |
| Adenoid cystic carcinoma |  | 30 | 3.3 |
| Salivary ductal carcinoma |  | 21 | 2.3 |
| Carcinoma in pleomorphic adenoma |  | 10 | 1.1 |
| Other tumors |  | 29 | 3.2 |

| TableS2. Distribution of different ultrasound devices in BPT and MPT | | | |
| --- | --- | --- | --- |
|  | The Philips iU22 | Esaote Mylab90 | Logic E9 |
| BPT | 159 | 224 | 264 |
| MPT | 58 | 92 | 110 |

Table S3. Results of univariate and multivariate analyses of the US characteristics of PTs in the training dataset

| Characteristic | Univariable analysis | | Multivariable analysis | |
| --- | --- | --- | --- | --- |
|  | Odds Ratio (95%CI) | P value | Odds Ratio (95%CI) | P value |
| Age(years) | 0.978(0.967,0.990) | ＜0.001 | 0.989(0.975,1.004) | 0.146 |
| Location | 0.761(0.557,1.039) | 0.085 | 1.422(0.913,2.215) | 0.120 |
| Shape | 0.263(0.188,0.367) | ＜0.001 | 0.633(0.404,0.994) | 0.047 |
| Margin | 0.028(0.015,0.049) | ＜0.001 | 0.052(0.027,0.101) | ＜0.001 |
| Posterior acoustic | 9.255(6.283,13.635) | ＜0.001 | 4.013(2.446,6.584) | ＜0.001 |
| Calcification | 0.299(0.163,0.549) | ＜0.001 | 0.831(0.318,2.174) | 0.706 |

| Table S4.Cohen Kappa’s agreement between Radiologists A and B | | | |
| --- | --- | --- | --- |
| Variable | ICC | 95% CI | P-value |
| Location | 0.842 | 0.807-0.877 | ＜0.001 |
| Shape | 0.902 | 0.803-0.931 | ＜0.001 |
| Margin | 0.938 | 0.796-0.882 | ＜0.001 |
| Echo | 0.811 | 0.740-0.882 | ＜0.001 |
| Cystic areas | 0.857 | 0.808-0.906 | ＜0.001 |
| Posterior acoustic enhancement | 0.947 | 0.922-0.972 | ＜0.001 |
|  |  |  | ＜0.001 |
| Calcification | 0.828 | 0.740-0.882 | ＜0.001 |

Table S5. Delong test of models in the internal-test cohort

|  | Resnet 18 | Resnet 50 | Vgg 11 | Vgg 16 | Mobilenetv2 |
| --- | --- | --- | --- | --- | --- |
| Resnet 18 | 1 | 0.018 | 0.017 | 0.010 | 0.001 |
| Resnet 50 | 0.018 | 1 | 0.746 | 0.497 | 0.004 |
| Vgg 11 | 0.017 | 0.746 | 1 | 0.353 | 0.027 |
| Vgg 16 | 0.010 | 0.497 | 0.353 | 1 | 0.010 |
| Mobilenetv2 | 0.001 | 0.005 | 0.027 | 0.010 | 1 |

| Table S6. Comparison between other references and this study | | | |  |  |  |  |  |  |  |  |  |
| --- | --- | --- | --- | --- | --- | --- | --- | --- | --- | --- | --- | --- |
|  | Image | Patients | BPT | MPT | Methods | Optimal model | AUC | ACC | SE | SP | PPV | NPV |
| This study | Conventional US image | 819 | 568 | 251 | Deep learning | Resnet18 | 0.947(0.915,0.979) | 0.885 | 0.782 | 0.927 | 0.811 | 0.914 |
| Zheng et al. ^[18]^ | CT images of three phases | 388 | 310 | 78 | Machine learning | SVM | 0.840 (0.761, 0.911) | 0.853 | 0.696 | 0.892 | 0.800 | 0.858 |
| Yu et al.* ^[19]^ | arterial-phase CT images | 573 | 396 | 177 | Deep learning | MobileNet V3 | 0.890 (0.844,0.937) | 0.846 | 0.828 | 0.860 | 0.716 | 0.917 |
| Gunduz et al ^[20]^ | multiparametric MRI | 123 | 99 | 24 | Deep learning | InceptionResNetV2 | none | 0.921 | 0.833 | 0.940 | 0.959 | 0.972 |
| Wang et al ^[36]^ | Conventional US image | 251 | 177 | 74 | Deep learning | EfficientNetB3 | 0.820 | 0.800 | 0.772 | 0.811 | 0.630 | 0.896 |
| *These metrics are the performance of the model on External-testing set. | | | | |  |  |  |  |  |  |  |  |
| AUC: area under the curve, ACC:Accuracy; SE: Sensitivity; SP: Specificity; PPV: positive prediction value, NPV: negative prediction value; BPT: benign parotid tumors; MPT : Malignant parotid tumors | | | | | | | | | | | | |


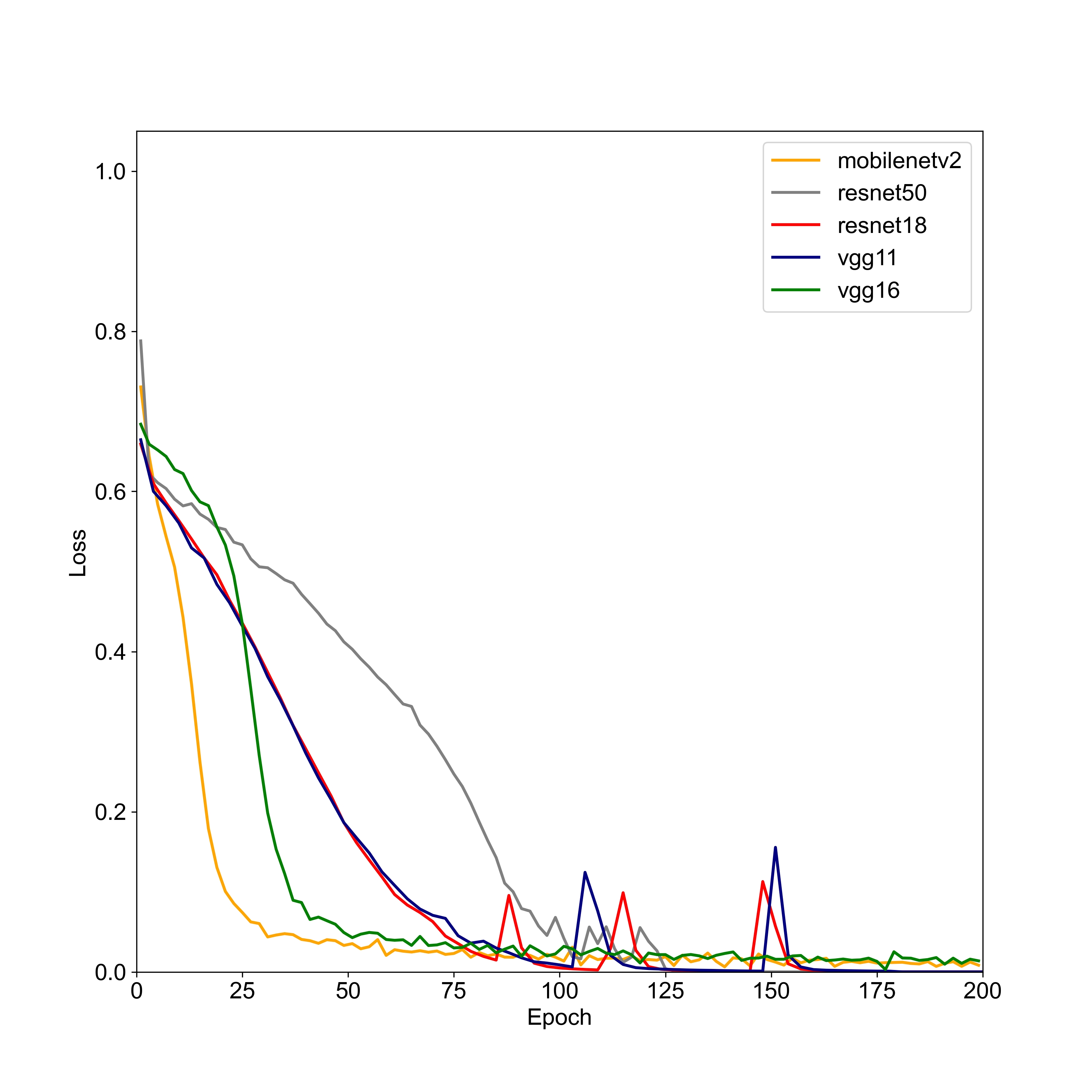


Figure S1. loss versus epoch during CNN model training and validation
